# Client and Clinician Experiences and Perspectives of Exercise Physiology Services During the COVID-19 Pandemic: Qualitative Study

**DOI:** 10.2196/46370

**Published:** 2023-12-21

**Authors:** Riley C C Brown, Shelley E Keating, Patrick J Owen, Paul S Jansons, Jenna McVicar, Christopher D Askew, Kelly M Clanchy, Ralph Maddison, Andrew Maiorana, Suzanne M Robinson, Niamh L Mundell

**Affiliations:** 1 School of Human Movement and Nutrition Sciences The University of Queensland Brisbane Australia; 2 Institute for Physical Activity and Nutrition School of Exercise and Nutrition Sciences Deakin University Geelong Australia; 3 VasoActive Research Group School of Health University of the Sunshine Coast Sippy Downs Australia; 4 Sunshine Coast Health Institute Sunshine Coast Hospital and Health Service Birtinya Australia; 5 School of Health Sciences and Social Work Griffith University Gold Coast Australia; 6 Menzies Institute of Health Griffith University Gold Coast Australia; 7 Allied Health Department Fiona Stanley Hospital Perth Australia; 8 Curtin School of Allied Health Curtin University Perth Australia; 9 Deakin Health Economics Institute for Health Transformation Deakin University Melbourne Australia; 10 Faculty of Health Sciences Curtin University Perth Australia

**Keywords:** telehealth, coronavirus, telemedicine, eHealth, rehabilitation, qualitative study, exercise, exercise physiology, clinician, engagement, interview, tool, implementation, effectiveness

## Abstract

**Background:**

The COVID-19 pandemic led to changes in the delivery of exercise physiology services. The lived experience of those who continued to provide or receive exercise physiology services during the heightened public health restrictions of the inaugural year of the COVID-19 pandemic has received little attention to date. Acquiring this knowledge will be fundamental in addressing whether telehealth is a viable option for service delivery in exercise care, research, and policy. This is especially pertinent in the wake of the COVID-19 pandemic and subsequent global interest in digital health delivery of health care services.

**Objective:**

This study aims to explore the clinician and client experiences and perspectives of exercise physiology services delivered in person or via telehealth during the inaugural year of the COVID-19 pandemic (after January 25, 2020; the date of the first confirmed case in Australia).

**Methods:**

Eligible participants for this study were adult (aged 18 years or older; capable of understanding and writing in English) clients who received and clinicians who delivered 1 or more exercise physiology sessions in Australia during the first year of the COVID-19 pandemic (June 2020 to June 2021). The data collection period spanned from January 20, 2021, to September 24, 2021. A total of 18 semistructured individual interviews were conducted with accredited exercise physiologists (n=7) and clients (n=11) who engaged with exercise physiology services during this period. All interviews were digitally recorded and transcribed verbatim. Thematic analysis was conducted with themes and subthemes derived using deductive and inductive approaches.

**Results:**

A total of 3 dominant themes, each with 2 subthemes, were identified. The first theme was that telehealth enables access to services but limits the use of some clinical tools. Remote access to services was valued by both clinicians and clients, but the exercise clinical environment could not be replicated over telehealth. This was especially true regarding access to exercise equipment. Second, engagement and the “relational space” are limited by telehealth. Perceived challenges regarding social interactions and a sense of community were a limitation for clients, and difficulties fostering clinician-client report were noted by clinicians. Finally, technological challenges are pervasive in the telehealth delivery of exercise services. Both clinicians and clients noted that systems necessary to facilitate telehealth frequently disrupted delivery, and client-based technical issues were influenced by digital health literacy.

**Conclusions:**

Shared client and accredited exercise physiologist experiences highlight key considerations for the ongoing implementation of telehealth to facilitate the uptake and effectiveness of exercise physiology services. These findings imply that the co-design of solutions to client-perceived limitations of telehealth delivery is warranted.

## Introduction

In response to the heightened public health restrictions in the inaugural year of the COVID-19 pandemic and associated physical distancing recommendations [1], a marked rise in the use of telehealth delivery for the provision of health care services has occurred worldwide [2-4]. Telehealth refers to remote health care engagement between a clinician and client via either a synchronous (real-time) or asynchronous (delayed) interaction [5]. From October 2019 (before the COVID-19 pandemic) to April 2020 (during the COVID-19 pandemic), telehealth delivery of hospital outpatient care in the United States increased 29-fold [6]. Similarly in Australia, a 24-fold increase in telehealth delivery of hospital outpatient care was observed from February 2020 (before the COVID-19 pandemic) to April 2020 (during the COVID-19 pandemic) [7].

The uptake of telehealth delivery in response to the COVID-19 pandemic extended beyond the hospital setting. Accredited exercise physiologists (AEPs) are university-qualified allied health professionals specializing in the individualized assessment and evidence-based delivery and prescription of exercise for a wide range of patient cohorts [8]. An audit of exercise physiology services in Australia showed that despite only 25% of clinicians offering some form of telehealth delivery prior to the COVID-19 pandemic, 91% of clinicians used telehealth delivery during the COVID-19 pandemic [5]. These data support the notion that clinicians who provided exercise physiology services were able to adapt and continue to do so during the COVID-19 pandemic. Previous literature examining the experiences of clients with disabilities undertaking allied health services via telehealth during this period revealed several barriers to implementation including difficulty establishing clinician-client relationships and reliance on caregiver facilitation [9]. However, the lived experiences of AEPs and their clients during this transition remain unknown. This is of particular importance given that only 52% of clients accepted the offer to receive exercise physiology services via telehealth delivery [5]. These experiences will provide valuable insight into AEP and client perspectives of telehealth service delivery. Along with previous literature, this may contribute to broader dissemination of telehealth across clinical exercise physiology services in Australia, including the co-design of exercise delivery options for optimal uptake.

This study aimed to explore the clinician and client experiences and perspectives of exercise physiology services delivered in person or via telehealth during the inaugural year of the COVID-19 pandemic (after January 25, 2020; the date of the first confirmed case in Australia). To enable a broader generalization of findings and application of recommendations, we sought to identify common themes among the experiences and perspectives of both AEPs and clients.

## Methods

### Study Design and Setting

This qualitative study was embedded within a larger study examining the use and effectiveness of exercise physiology services in Australia before and during the COVID-19 pandemic [5]. We used a qualitative description approach [10] to capture the client and AEP perspectives and experiences of exercise physiology services during the inaugural year of the COVID-19 pandemic (after January 25, 2020; the date of the first confirmed case in Australia).

### Participants

Eligible participants for this study were adult (aged 18 years or older; capable of understanding and writing in English) clients who received and clinicians who delivered 1 or more exercise physiology sessions in Australia during the first year of the COVID-19 pandemic (June 2020 to June 2021). Purposive sampling was used to identify potential participants from a recent retrospective audit of AEP practices [5]. Participants were primarily sought through social media advertisements and correspondence released by the professional accreditation body Exercise & Sport Science Australia. Word-of-mouth referrals were obtained through the networks of study authors. Participants from all Australian states and territories were invited to participate. Potential participants were initially contacted via email by the project manager. Collectively, 9% (7/80) of clinicians and 8% (11/138) of clients agreed to participate. Recruitment ceased on the assessment by the interviewer that data saturation was reached. No reasons for refusal to participate were recorded and no participants withdrew consent following enrollment.

### Data Collection

The data collection period spanned from January 20, 2021, to September 24, 2021. A total of 18 individual semistructured interviews were conducted via videoconference in the participants’ choice of setting (eg, home or workplace). A single interviewer (PJ; PhD, research fellow and AEP, and male) conducted all interviews. There were no established relationships between the interviewer and participants prior to study commencement. The interviewer introduced themselves as a member of the research team and disclosed all relevant study-based information regarding aims, funding, and participation.

Semistructured interviews followed an interview guide that listed a number of key questions developed by the interviewer (Multimedia Appendix 1). When necessary, the interviewer asked further questions to clarify or obtain additional information following responses to key questions. No repeat interviews were conducted, and all interviews were digitally recorded and transcribed verbatim by an external commercial transcription company (TranscribeMe). No field notes were made during or after the interviews. The mean (SD) duration of all interviews was 12.9 (3.3) minutes. Due to financial limitations, transcripts were not returned to participants for comment and correction.

### Data Analysis

Independent thematic analysis was formally conducted by one researcher (RCCB) using Microsoft Excel (Microsoft). These data were validated by another researcher (SEK). Each researcher independently familiarized themselves with all data and then extracted, coded, and tabulated meaningful sentences or phrases. Themes and subthemes were derived using deductive and inductive approaches and compared. These were guided by the approach of Braun and Clarke [11]. Consensus discussions were held digitally to consolidate themes and subthemes. When a consensus was not clear, a third researcher (PJ) arbitrated decisions on final themes. The trustworthiness of the data was assessed using four criteria: (1) credibility (data triangulation from multiple perspectives; with 2 researchers, RCCB and SEK, confirming themes), (2) transferability (rich details on the study methods with purposive sampling of participants), (3) dependability (data triangulation by 2 researchers, RCCB and SEK, with rigorous data collection techniques, procedures, and analyses), and (4) confirmability (debriefing, reflexivity, and meetings between RCCB, SEK, and NLM were conducted to establish analytic rigor). Dominant themes were cross-checked with exemplar quotes to confirm consistency. No participants were asked to provide feedback on the findings. Data saturation for both AEPs and clients was achieved prior to ceasing recruitment. Data saturation was determined when little or no new information was being garnered from interviews to address the research question [12,13].

### Ethical Considerations

The study was conducted in line with the National Statement on Ethical Conduct in Human Research (2007) and the Declaration of Helsinki. It was approved by Deakin University Human Ethics Advisory Group Health (90-2020-200512), and the findings were reported in accordance with the Consolidated Criteria for Reporting Qualitative Research (COREQ) [14]. All participants provided written informed consent prior to involvement in the study. Informed consent covered sensitive and private information. Participants engaged voluntarily in the research project and were able to opt up at any time. All participant data were deidentified prior to theme derivation.

## Results

### Overview

**Figure 1:**
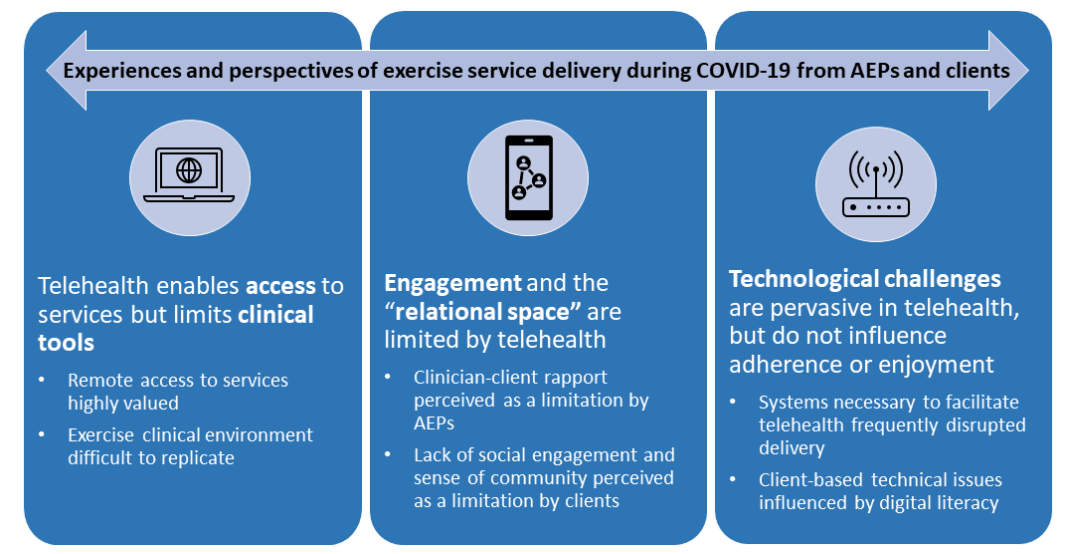
Pertinent themes and subthemes extracted from interviews regarding exercise physiology telehealth delivery during the COVID-19 pandemic. AEP: accredited exercise physiologist.

Client and AEP demographics are shown in Table 1. A total of 11 clients and 7 AEPs participated in interviews, and 3 key themes with pertinent subthemes were identified (Figure 1 and Table 2). Experiences regarding overall access to exercise physiology services during the COVID-19 pandemic were often shared by both AEPs and clients.

**Table 1:** Demographics of clients and accredited exercise physiologists (AEPs).

Characteristics		Clients (n=11)		AEPs^a^ (n=7)
**Sex, n (%)**				
	Female	5 (45)	2 (29)	
	Male	6 (55)	5 (71)	
**State or territory of residence, n (%)**				
	Victoria	11 (100)	3 (43)	
	New South Wales	0 (0)	2 (29)	
	Queensland	0 (0)	2 (29)	
**Highest level of education, n (%)**				
	Secondary	2 (18)	N/A^b^	
	Tertiary	9 (82)	7 (100)	
**Current employment status, n (%)**				
	Full-time	2 (18)	5 (71)	
	Part-time	3 (27)	2 (29)	
	Unemployed	2 (18)	N/A	
	Retired	4 (36)	N/A	
**AEP role, n (%)**				
	Salary (private clinics or companies)	N/A	5 (71)	
	Sole trader	N/A	1 (14)	
	Not for profit	N/A	1 (14)	
AEP years of practicing, mean (SD)		N/A		7.9 (3.4)

^a^AEP: accredited exercise physiologist.

^b^N/A: not applicable.

**Table 2:** Themes and subthemes with illustrative quotes.

Themes and subthemes		Example illustrative quote (AEPs^a^)		Example illustrative quote (clients)
**Telehealth enables access to services but limited use of some clinical tools**				
	Remote access to services valued	“I think it’s really cost-effective because you are paying you’re—let’s say you’re at home. You’re paying your electricity, and you’re paying Wi-Fi, and laptop. Usually, you have a laptop anyway, so. And for clients, also cost-effective.” [AEP #4] “I would say flexibility. So you could work from home. You could work at different times than you normally would. Also the flexibility for the client. They quite like that.” [AEP #7]	“I mean, it’s definitely accessible. It’s not like it’s limited to certain people. It’s not something that requires a lot of cost. Most people have phones or computers or tablets or whatever that have the capabilities to access Zoom or other similar programs. I mean, it certainly provides easy access to even sometimes specialists and stuff like that, not even necessarily something that I would do on a weekly occasion or a couple of times a week.” [Client #6] “I think it would be very cost-effective. I don’t know, I would imagine it depends. Cost-effective from a health system perspective or from a consumer’s perspective? I’m not sure. Certainly from a consumers perspective if you’re not having to pay travel costs and that sort of thing, then yeah.” [Client #11] “I don’t have to worry about getting there or making sure—and no, I’m not one of these people who runs chronically late, but you don’t have to worry about getting there on time.” [Client #1] “There’s no way I won’t wake up at 9 o’clock to do my Telehealth...Whereas I would cancel if I had heaps of work to do face to face.” [Client #2]	
	Exercise clinical environment cannot be replicated	“I think we got creative...some of the games and screen sharing and songs and integrated dance programs and stuff that we ended up doing were advantages of telepractice.” [AEP #1] “It would be harder to give them probably what you think is the best...for them, obviously, as opposed to them coming in to the clinic or if they had equipment...limited.” [AEP #2] “It’s just the inability to assess and to prescribe and to pick up on things with clients. For instance, I held a Telehealth class today, and we had new participants in the morning, and it was just very hard for me to kind of be like, “Can you show me that exercise?” or, “Can you show me the range of your hip?” sort of thing. So just not being able to physically do things was probably the biggest negative.” [AEP #6]	“I’m kind of limited with the equipment that I have at home, it is somewhat limiting in what I can do with the exercise program that I’ve got because I’m relying a lot on the equipment that I can use and stuff that I’ve got access to, which is a lot less than what would be available compared to the clinic.” [Client #6] “Possibly, explanation of exercises, trying to get the—especially some of the more complicated ones, where there was a series of movements in the one exercise using different equipment. Trying to get it right, that took more time which was—I mean, they got very near. Or I got very near. That’s possibly a negative, time it took to explain the complexity of some exercises.” [Client #7]	
**Engagement and the “relational space” are limited by telehealth**				
	Clinician-client rapport perceived as a limitation by AEPs	“Yeah. I think that’s always going to be just the interpersonal barrier, so the best way to build up rapport in a relationship is probably face to face.” [AEP #5] “Engaging clients and keeping them engaged in programs and technology was a huge one.” [AEP #1]	N/A^b^	
	Lack of social engagement and sense of community perceived as a limitation by clients	N/A	“You have...a few jokes, a bit of social chit-chat in between sets and stuff. And that’s as good as it will get...and the gap is the relationship in that space on the face-to-face is—again, it’s just about human beings being together and chatting and engaging. And, as I said, there’s a community in the clinic context. There’s the supervising instructor or clinician, and then there’s other students and other clients stationed. So it’s a little more social.” [Client #3] “It’s a bit less personal because, again, you’re talking to your screen, although the person’s face is on the other—is there, you’re not there with the person.” [Client #6]	
**Technological challenges are pervasive in the telehealth delivery of exercise services**				
	Systems necessary to facilitate telehealth frequently disrupted delivery	“Then sometimes, it would glitch as well, which would hold thing up, and they’d [sic] get a little bit annoyed by it and not happy with it, so it could affect the way the session ran.” [AEP #7] “So actually logging into a computer is quite challenging for a lot of people. Using all that assisted technology and it’s another step to engage.” [AEP #1]	“During the session, the instructor, they freeze so all you’ve got is an audio.” [Client #2] “So when trying to do it by, say, me taking a video myself walking, so am I checking out my gait. The problem [inaudible] you can’t send a video by email because it’s much too—it’s too big a file. That’s the problem. So we haven’t got around that one.” [Client #4]	
	Client-based technical issues influenced by digital literacy	“Understanding technology...patients can have that issue.” [AEP #5] “We had a lot of people that didn’t have access to devices...(they) can’t use devices independently.” [AEP #1]	“My equipment could be better. It could be whatever, but well, we usually work around it and then we solve it. Which is good.” [Client #4]	

^a^AEP: accredited exercise physiologist.

^b^N/A: not applicable.

### Telehealth Enables Access to Services but Limited the Use of Some Clinical Tools

#### Remote Access to Services Valued

Telehealth-enabled access to exercise physiology services beyond the usual in-person health care setting was valued by both AEPs and clients. For the clients, this related to the convenience of home-based sessions that reduced travel burden (notably time and cost), while for the AEPs, this related to the capacity to provide their services to a wider geographical range.

Accessibility, ability to be able to service patients in remote areas.

AEP #5

Accessibility for me. Being able to do it in the morning pretty much from home in the space that I’ve set aside. The benefit is that it’s less travel.

Client #3

A difference in opinion on the delivery of telehealth exercise physiology services was noted. AEPs preferred in-person modalities and perceived that clients also preferred this method. Clients described a preference for a hybrid approach using both in-person and telehealth to improve exercise motivation.

Oh, I prefer face to face (for delivery of services)

AEP #6

Overall, I think people’s eagerness to come back on-site kind of shows that they do prefer face-to-face.

AEP #1

I’m happy to stay with Telehealth and go in every two weeks, but face to face. That is probably how I would probably work it.

Client #1

No, I don’t have a preference, I just have—there’s a different style of engagement for me so that’s the only thing that it lacks on, which one I will do. This week I’ve got telehealth, then face-to-face and telehealth before the end of the week, and I’m really enthused about both.

Client #3

#### Exercise Clinical Environment Cannot Be Replicated

Both clients and AEPs described challenges and limitations due to the lack of access to the range of dedicated aerobic and resistance training equipment typically used in usual in-person settings. Clients described the home environment as not “purpose built” for the provision of exercise physiology services, particularly for resistance training. This made it difficult to replicate loading, attain higher intensity prescriptions, and limit exercise range. AEPs reported supporting clients to find innovative ways to replicate exercises in their home environment.

...when people come see us in the clinic, we’ve got equipment..., whereas when you’re doing it at home, you’re like [sic], “Okay, do you have a tin of tomatoes?” or, “Do you have a step? What sort of space do you have? Do you have something you could hold onto?”

AEP #6

And the other thing’s getting on bikes, getting on cardio machines (at a clinic) as opposed to just using your own body weight and resistance (at home).

Client #3

The inability to seamlessly integrate the assessment and monitoring of important clinical outcome measures remotely that are commonly implemented during in-person delivery of exercise physiology services was noted by both AEPs and clients. Telehealth restricted the ability to use visual and audio cues for the expert analysis of exercise techniques that challenged comprehension. The outcome assessment of objective clinical measures (such as blood pressure, anaerobic capacity, and blood glucose) was also reported to be challenged or unavailable by telehealth. AEPs reported that being more familiar with the in-person or clinic environment resulted in less setup time and improved scheduling capability. However, the risk of COVID-19 transmission was highlighted as a key negative to in-person delivery that necessitated telehealth. Collectively, perceptions from both AEPs and clients were that telehealth-delivered programs were less effective.

It’s hard doing things like some of the sort of blood pressure, heart rate, ECG, the blood glucose, those sorts of things, hard doing that sort of through telehealth.

Client #11

In terms of initial assessments, yes. Just in terms of having standardised—for example, for anaerobic tests, not having a treadmill or a bike obviously makes it a bit more difficult, so you’re relying a lot more on the subjective. This subjective stuff’s fine, but you’re relying a lot more on the subjective side of things than you would be on the objective side of things.

AEP #2

### Engagement and the “Relational Space” Are Limited by Telehealth

#### Overview

Perceived challenges regarding social interactions, and the lack thereof, during exercise sessions were shared by both AEPs and clients. However, the point of emphasis differed, with clients raising issues with social engagement and AEPs the ability to foster rapport.

#### Clinician-Client Rapport Perceived as a Limitation by AEPs

The ability to forge and foster clinician-client rapport was commonly perceived as a limitation by AEPs. This led to a perceived suboptimal working relationship between AEPs and clients. Notably, some AEPs felt that technology hindered their ability to communicate as they would in person.

And a bit harder to kind of build rapport or relationships over the telephone or video link.

AEP #5

I just found that conversation being slower as well as it normally would in a face-to-face session, which sometimes because of that I found session would run a little bit shorter than they normally would.

AEP #7

#### Lack of Social Engagement and Sense of Community Perceived as a Limitation by Clients

The lack of social engagement with staff and others, and of being part of a group or community, was perceived as a more pertinent limitation by clients. This negatively affected their enjoyment of exercise services, where personal relationships with professionals and peers were valued.

The relational space is different, and I think the downside of telehealth is the [inaudible] to sort of see and be alongside in the same way as face-to-face. And I personally enjoyed the richness of the encounter with the trainer, the therapist, the clinician, and that’s missing.

Client #3

There’s not really much social interaction apart from with your trainer.

Client #8

### Technological Challenges Are Pervasive in Telehealth Delivery of Exercise Services

#### Overview

Experiences reported by both AEPs and clients regarding technology tended to focus on negatives at either the system (connectivity) or operator (digital literacy) level.

#### Systems Necessary to Facilitate Telehealth Frequently Disrupted Delivery

Telehealth systems necessary to facilitate the provision of exercise physiology services tended to be perceived as challenging by both AEPs and clients. Notably, factors were often perceived as outside the control of either the AEP or the client. Both AEPs and clients shared experiences of predominantly client-based technical issues. However, these issues were generally rectified with troubleshooting.

Sometimes connectivity issues.

AEP #5

The initial barrier is the technology side of things. Just taking time to work out whether it’s going to be Zoom or phone or video or phone, and getting people to work out how to do it with older clients that I often work with.

AEP #3

It’s something with my—one of my devices decided it didn’t want to—either the audio or something didn’t work, so we just hooked up a different device and it worked fine...And technology does that of its own accord whatever happens.

Client #1

Yeah, only once or twice. I think when I thought, “I’ll try my Surface Pro,” I hadn’t—I’m not too bad with technology, but that didn’t work, and we just flicked over back to the phone, and that was fine.

Client #5

#### Client-Based Technical Issues Influenced by Digital Literacy

Higher perceived digital literacy among clients predicted a better ability to overcome any issues with technology. This included having access to the required technology, using technology, and being able to manage technological issues as they arose.

One of the big negatives were a lot of my clients were elderly people, so there was difficulty using the technology.

AEP #7

We’re reasonably technology literate, so that worked pretty well for us, but I would imagine others that are less technology literate would struggle a little.

Client #9

## Discussion

### Principal Findings

The three main themes identified in this study of AEP and client experiences and perceptions of exercise physiology services during the COVID-19 pandemic were as follows: (1) telehealth enables access to services but limits the use of some clinical tools, (2) engagement and the “relational space” are limited by telehealth, and (3) technological challenges are pervasive in telehealth delivery of exercise services. Positive experiences included increased geographical reach and time or cost efficiency. Negative experiences stemmed from accessibility to equipment necessary for completing prescribed exercises and assessing or monitoring clinical outcomes, challenges with clinician-client rapport, inferior social engagement with peers and practitioners, and the presence of technological issues (system- and operator-based). This study adds to the current literature by providing insights into AEP and client perspectives on telehealth service delivery. This is especially important in the wake of the COVID-19 pandemic, where interest in digital health has been amplified.

We identified key positive themes relating to exercise physiology service provided during the COVID-19 pandemic. Specifically, both AEPs and clients acknowledged that telehealth delivery of services increased geographical reach and tended to be more cost- and time-efficient than in-person delivery. A 2021 report of Australian clients who used telehealth allied health services during the COVID-19 pandemic also identified positive experiences regarding time efficiency and increased geographical reach, yet conversely reported that multiple clients considered telehealth delivery poor value for money compared to in-person delivery [15]. Inconsistency regarding experiences of cost-efficiency likely stems from examining allied health services collectively, whereby hands-on treatments, such as manual therapy and podiatry services, are physically incompatible with telehealth delivery [15,16]. An analysis of financial costs associated with Australian exercise physiology services during the COVID-19 pandemic indicated that across a mean (SD) treatment duration of 11 (7) weeks, the cost of exercise physiology services solely delivered via telehealth was Aus $89.91 (US $59.71) less than that delivered in person only [17]. These apparent differences cost should be interpreted with caution, however, given the omission of effectiveness data and the disproportionate representation of National Disability Insurance Scheme clients. The positive experiences identified in this study support the notion that the continued use of telehealth delivery methods during the COVID-19 pandemic may benefit the provision of exercise physiology services due to remote access to services being valued. Accessibility to patient-centered health care is a key strategic imperative for the Australian government [18]. These results may indicate the value of telehealth exercise physiology services for the promotion of patient-centered health care.

Several negative experiences of exercise physiology services provided during the COVID-19 pandemic were also identified. Challenges pertaining to equipment accessibility, social engagement, and the use of technology were commonly identified by both AEPs and clients. Interestingly, the safety of telehealth delivery was not listed by clients or AEPs as a barrier to implementation. This finding contrasts with previous literature analyzing allied health professionals’ perceptions of telehealth exercise services, where safety and suitability of clientele were raised as a key consideration prior to implementation [19,20]. The challenges identified in this study were similar to those of a 2021 report of Australian clients and clinicians who used allied health services during the COVID-19 pandemic [9]. These include difficulties conducting some clinical assessments via telehealth and difficulties fostering the client-clinician relationship [15]. Furthermore, a recent qualitative analysis of telehealth allied health service delivery for clients with permanent disabilities reported similar challenges [9]. These challenges included inhibition of clinician feedback, difficulty fostering client-clinician rapport, and lack of access to resources [9]. Addressing challenges associated with the telehealth delivery of exercise physiology services is of known importance to facilitate uptake and effectiveness. For example, a recent systematic review examining the efficacy of exercise training for patients with chronic disease provided via telehealth delivery observed access and usefulness of technology as the main drivers of patient satisfaction [21]. Furthermore, both AEPs and clients perceived exercise physiology services via telehealth delivery as less effective than in-person delivery. This contrasts with recent findings determining that videoconferencing exercise interventions are at least as effective as in-person services for the improvement of exercise capacity and quality of life [21]. However, the context of videoconferencing exercise interventions included in this review differs from this study, with studies included being subjected to highly controlled clinical environments with objective data. This is fundamentally different from clinical exercise practice adapting quickly to COVID-19 pandemic–related public health restrictions.

The lack of social engagement expressed by clients in this study appeared to be influenced by the one-on-one nature of telehealth exercise physiology sessions delivered. Group-based videoconferencing may address some aspects of this issue but have additional considerations for delivery prior to implementation. While AEPs unanimously preferred in-person delivery and perceived that clients also preferred this modality, clients conveyed mixed opinions regarding which modality was superior for motivation to attend sessions. This suggests that there are a variety of factors that may influence modality preference (eg, time and digital literacy). Given the importance of a client-centered approach for achieving clinical outcomes, the value placed on a flexible mode of delivery by clients should be considered when designing exercise programs. This finding demonstrates the importance of co-designing exercise interventions between AEPs and clients for optimal uptake. Together, our observations and those from prior studies suggest that a range of barriers should be considered when using telehealth delivery of exercise physiology services.

The results of this study warrant consideration for ongoing and future exercise physiology telehealth services. To improve the reliability and quality of service, AEPs should ensure that universal telehealth requirements are met [22]. Of particular importance is the development of a skilled workforce competent in the administrative and operational aspects of telehealth practice. This includes the formulation of telehealth action plans (including strategies for issues with technology) for implementation by both administrative and clinical staff and the use of exercise assessment or prescription methods that lend themselves to delivery via telehealth. Examples may include validated patient-reported outcome measures and the application of telehealth-appropriate exercise programming that aligns with client goals and preferences. Further suggestions to improve practice include establishing expectations of sessions and access to support staff to facilitate telehealth sessions [9]. Prior to facilitation, evaluation processes should also take place on an individual level to assess client suitability for telehealth and preference for service modality [9,23]. Importantly, the co-design of solutions to client-perceived limitations of telehealth is warranted. Given that nationally funded AEP telehealth service uptake through Australia’s universal health care system (Medicare) is considerably low [24], and the government’s strategic imperative to provide more accessible patient-focused health care, the results of this study and consequent recommendations for practice should be considered prior to service implementation.

This study was strengthened by the inclusion of both AEPs and clients, which increased the ecological validity and generalizability of the lived experiences identified. The use of 2 independent data analysts who did not conduct the semistructured interviews further reduced the risk of bias during data analysis. The unique findings of this study complement previous literature, potentially contributing to the uptake and dissemination of telehealth in exercise services in Australia. The limitations of this study, however, should also be considered. First, all participants were recruited from the state of Victoria. This occurred even though participants from all states and territories were invited to participate. Second, financial restrictions precluded the return of transcripts and findings to participants for corrections and interpretation, respectively. As member checking is a component of assessing credibility, this has implications for the trustworthiness of the transcribed data and should be interpreted with caution. Third, the questions included in the interview guide of this study were designed to examine lived experiences and perspectives, rather than specifics regarding acceptability, barriers, and facilitators; therefore, future qualitative studies are necessary to evaluate and draw conclusions on these specific components of exercise physiology services during the COVID-19 pandemic.

### Conclusions

The evaluation of perceptions and experiences of AEP and client lived experiences of exercise physiology services during the COVID-19 pandemic showed that despite pervasive technological challenges, telehealth delivery enabled access to services. Clinician-client rapport, social engagement, and the capacity to use dedicated equipment for exercise prescription and outcome assessment were viewed as negatives to telehealth service delivery. These findings imply that the co-design of solutions to client-perceived limitations of telehealth delivery is warranted. Shared client and AEP experiences highlight key considerations for the implementation of telehealth delivery into exercise physiology services to facilitate uptake and effectiveness.

## Appendix

Multimedia Appendix 1 Interview question template for clients and accredited exercise physiologists.

## Abbreviations

AEP: accredited exercise physiologist

## Data Availability

The data sets generated and analyzed during this study are available from the corresponding author on reasonable request.
