# Supplementary material for: Client and Clinician Experiences and Perspectives of Exercise Physiology Services During the COVID-19 Pandemic: Qualitative Study
Source: J Med Internet Res. 2023 Dec 21;25:e46370. doi: 10.2196/46370 (PMC10767626; doi:10.2196/46370)
Supplement: Multimedia Appendix 1 [file jmir_v25i1e46370_app1.docx]

**Multimedia Appendix 1.** Interview question template for clients and accredited exercise physiologists (AEP).

| Participant | Interview question |
| --- | --- |
| Client | 1. What have you found the benefits of attending the face-to-face program to be? |
|  | 1. What have you found the negatives of attending the face-to-face program to be? |
|  | 1. What have you found the benefits of attending the telehealth program to be? |
|  | 1. What have you found the negatives of attending the telehealth program to be? |
|  | 1. Do you prefer either telehealth or face-to-face prescribed programs for your ongoing exercise physiology consultations, and why? |
| AEP | 1. What have you found the benefits of delivering a face-to-face program to be? |
|  | 1. What have you found the negatives of delivering the face-to-face program to be? |
|  | 1. What have you found the benefits of delivering the telehealth program to be? |
|  | 1. What have you found the negatives of delivering the telehealth program to be? |
|  | 1. Do you prefer either telehealth or face-to-face delivered programs for your ongoing exercise physiology consultations, and why? |
